# Injuries among young workers in career-technical-vocational education and associations with per pupil spending

**DOI:** 10.1186/s12889-018-6099-9

**Published:** 2018-10-20

**Authors:** Derek G. Shendell, Saisattha Noomnual, Jesse Plascak, Alexsandra A. Apostolico

**Affiliations:** 1 Environmental and Occupational Health And New Jersey Safe Schools Program (NJ SS) Rutgers School of Public Health, 683 Hoes Lane West, 3rd Floor SPH Building, Piscataway, NJ 08854-8020 USA; 2 University of Illinois-Chicago, Chicago, USA; 3 Department of Epidemiology, Rutgers School of Public Health, Piscataway, USA; 4 School of Public Health, The University of North Carolina, Chapel Hill, USA

**Keywords:** Adolescents, High schools, Injury, Per pupil spending, Secondary schools, Young workers

## Abstract

**Background:**

New Jersey Department of Education (NJDOE) requires by law for accidents/incidents (injury) involving career-technical-vocational education (CTE) students and staff to be reported within five business days to the NJ Safe Schools Program (NJSS) using an online surveillance system. NJ public schools and charter schools (CS) through school districts (SD) or county offices report school data annually to NJDOE, including per pupil spending (PPS). In this study, we examined potential associations of PPS with several variables on injury in NJ: injury cause, injury location on the body, injury type, injury severity, use of PPE, and location of treatment for injury.

**Methods:**

PPS data for December 1998–June 2015 from CTE SDs (one per NJ county, *n* = 21), four CS SD and eight county special services districts were analyzed. T-test examined potential differences in PPS regarding injury severity and use of personal protective equipment (PPE). Stepwise logistic regression assessed potential associations between PPS and various injury surveillance variables.

**Results:**

There were more CTE injuries reported among SD with lower PPS than among SD with higher PPS. Relatively less severe injuries, e.g., bruise/bumps and cuts/lacerations, more often occurred at schools and SD with higher PPS. Conversely, relatively more severe injuries, e.g., fractures, more often occurred at schools and SD with lower PPS.

**Conclusion:**

Future research should further investigate disparities regarding younger worker injuries reported within school-based career-technical-vocational education programs by PPS and other factors like sex or gender, severity, safety training provided and work experience at time of injury.

## Background

Unintentional injuries among adolescents and young adults age 21 and younger, an already susceptible, vulnerable subpopulation, are ongoing public health concerns. Approximately 18.1 million young workers under age 24 comprised about 13% of the workforce in the U.S. in 2013. [1] In 2012, 375 young U.S. workers died from work-related injuries. [1] In 2009, there were approximately 26,500 emergency-department (ED) treated illnesses and injuries among 15–17 year-old youth workers in the U.S., and it was estimated by the U.S. Centers for Disease Control and Prevention-National Institute of Occupational Safety and Health (NIOSH) only about one-third of incidents were even treated at hospitals. [2] Furthermore, in 2009, 4380 illnesses and injuries among workers under age 18 in the U.S. required at least one day away from work; this number excluded workers at small farms, local agencies and if self-employed. [2] Compared to older adults, these higher numbers of young worker injuries may relate to their inexperience, lack of safety and health knowledge and awareness due to a lack of proper training, cultural and economic barriers, and their biological and physiological characteristics, e.g., inadequate strength and cognitive skills to operate some potentially hazardous manual and automatically operated equipment for certain tasks. [1, 3, 4]

Secondary school resources such as per pupil spending (PPS), class size, teachers (e.g., numbers or teacher-to-student ratio), and the quality and content of curriculum are factors potentially influencing safety and health outcomes among students; several of these factors also relate to socioeconomic status (SES) indicators. Previous studies have suggested childhood injury outcomes, in terms of morbidity and mortality, varied by SES. Among students in supervised, school-sponsored career-technical-vocational education (CTE) programs, SES may influence reported injuries. [5] However, at present, few data exist on potential associations between PPS and injury and illness—overall or work-related—among secondary school students.

PPS has been defined to include money from federal, state, and local sources, and has varied among states, ranging from the lowest PPS state, Utah ($6555) to the highest, New York ($19,818); New Jersey was among higher PPS states ($17,572). [6] Recent research suggested adolescents attending schools with higher PPS had increased scores on the adult health utility index, and college students in the top quartile of PPS had a higher adult utility index score than those in the bottom quartile of PPS. [7] These findings also were supported by sibling fixed models, which suggested individuals who attended schools with higher PPS have better subsequent health outcomes than their siblings who attended schools with lower PPS. [7] Another study reported increased PPS was associated with decreased physical assault among educators. [8] Another study suggested U.S. schools with higher PPS more often prohibited use of physical activity as punishment in physical education. [9] Furthermore, in those higher PPS schools, nurse-to-student ratios and physician-provided services to students at school were higher, and they were also more likely not to offer brand-name fast food to students. [9]

Other studies besides those on PPS have suggested SES was an important factor for getting injured in general, whether non-fatal or fatal injuries, in particular among adolescents and young adults. [10] It must also be noted non-fatal injuries can be severe; youth workers can have permanent disabilities and work restriction. [11] Research also suggests higher parental SES was significantly associated with decreased work-related injuries among adolescents. [12] However, data are limited on associations between SES indicators, including PPS, and work-related injuries among adolescents and young adults, particularly on students enrolled in CTE programs.

CTE programs offer a great opportunity to prepare students—adolescents and young adults—to enter the work force, and encompass youth who could drop out from traditional schools. There are over 20,000 CTE and “Ready to Work” programs in the U.S. financially supported by the federal government (over $1 billion). [13] Moreover, students in CTE programs have been more likely to report having been informed of legal rights and having received safety training than those working outside of these structured programs. [14] The U.S. Office of Vocational and Adult Education previously estimated, on average, every high school student has taken at least one CTE course, and 1-in-4 students completed three or more courses in a single program area. [13]

Based on NJ Administrative code 6A:19–6.5, the New Jersey Department of Education (NJDOE) requires by law for accidents/incidents (injury or illness) involving CTE students and/or staff treated by a licensed physician, physician’s assistant, or advanced practice nurse to be reported to the NJ Commissioner of Education. [15–17] Incidents are directly reported to the NJ Safe Schools Program (NJSS) online surveillance system (via Psychdata) for aggregate analyses.

Data on incidence of injuries reported from CTE schools in NJ between 12/1998–12/2013 were described in a prior paper examining associations with District Factor Groups (DFG) scores as one potential indicator of area-level SES. [5] In the present study, we examined potential associations between injuries reported from students enrolled in NJ CTE schools and school district (SD)-level PPS. Although PPS is a SES indicator commonly used throughout the U.S., no known study has simultaneously tested whether DFG scores or SD-level PPS both are associated with injury characteristics occurring within CTE schools. Moreover, PPS is a more specific measure of SES than DFG scores. The DFG scores are derived from U.S. Census-based sociodemographic variables, but PPS is a direct reflection of monetary resources spent within each SD.

Specifically, in this paper, we examined potential associations of PPS with several key variables: injury cause, injury location on the body, injury type, injury severity, use of PPE, and location of treatment for injury. We hypothesized lower PPS school districts would have relatively more severe injuries, use PPE less often, and have higher number of reported injuries treated at hospitals/ED compared to SDs with higher PPS. In the present analyses, data collected through the NJSS incident reporting surveillance system between the years 12/1998–06/2015, i.e., 1.5 years more data than prior analyses, [5] were used along with PPS data from CTE and charter schools (CS) in NJ for state fiscal years 1998–2015. [18]

## Methods

Aggregate, de-identified injury surveillance data were used; there was no personal, identifying information. The Rutgers University-New Brunswick Institutional Review Board (IRB) has approved NJ Safe Schools Program incident surveillance and training-related evaluation activities as exempt research since they are based on various laws (IRB #021997 W0383). Overall, most injury reports were students (96%), with the remaining 4% of injuries being acquired by school staff (adults); these reports were excluded in further analyses.

PPS data were abstracted from a NJDOE database [18] and were expressed with different terms throughout the State of NJ Fiscal Year (FY) records as Total Cost Per Pupil (1999–2002), Total Comparative Cost Per Pupil (2003–2010), Budgetary Per Pupil Cost (2011-present), and Total Spending Per Pupil (2011-present). The definition of each term was similar, and served an indicator to allow comparisons of SD costs. The SD cost included a SD’s general fund and special fund budgets related to services for enrolled students. Other costs included in these indicators were costs of governance, support, and instruction considered common and generally uniform, i.e., staff salaries and fringe benefits, textbooks, supplies and materials, rentals and insurance, legal fees and other purchased professional, technical and property services. The former three indicators are different from the Total Spending Per Pupil. The Total Spending Per Pupil also includes state expenditures (i.e., pensions and social security payments) on behalf of the SDs; transportation costs (including costs for students transported to non-public/private and CS); and, legal judgments against the SD. In addition, Total Spending Per Pupil includes food services expenditures, including those covered by school lunch fees; capital spending budgeted in the general fund (facilities and equipment); special revenues supported by local, state, and federal revenues; payments by one SD to other private and public SD for the provision of regular, special, and preschool education services; school departments; and, an estimate of the SD’s share of the debt service the State of NJ is paying for school construction bonds issued for school construction grants and School Development Authority Projects. In this study, we did not use the Total Spending Per Pupil because data were only available 2011–2015. Instead, in this study, the indicator used was PPS, i.e., reflecting Total Cost Per Pupil, Total Comparative Per Pupil Cost, or Budgetary Per Pupil Cost (similar definitions). PPS of each SD for the 1998–2015 school/academic years (state fiscal years) were managed in Microsoft Excel by school types-- secondary schools of grades 7–12 or 9–12, county special services SD (CSSD), CTE SD, or charter school (CS) SD. Inflation adjustments to PPS were calculated using monthly consumer price index data specific to the education industry, averaged according to the academic fiscal year (July 1 – June 30), and standardized to 2010 U.S. dollars. [19] To investigate the association between annual PPS and work-related high school injuries, reported injuries were matched to each school’s PPS (based on its SD) by academic year.

As detailed elsewhere, [5] the year 2000 DFG scores were averaged at the county level and linked to each county/SD’s CTE program. This was necessary because despite each CTE program being reported separately as approved within a city/town SD or the county-wide service area, it could not receive a separate DFG scores because any student within the county (as opposed to geographically based local SD within a county) could attend the respective county-based CTE SD or special services SD.

### Data analysis

The distributions of mean PPS of CTE SDs and CTE plus non CTE SDs (i.e., eight CSSD and four CS SDs) were slightly right-skewed. In this study, PPS was categorized into higher PPS and lower PPS based on above and below the 50th percentile, respectively.

Statistical analyses were conducted within SPSS 24 (IBM) and SAS 9.4 (Cary, North Carolina). Data were not normally distributed and were log transformed. T-test assessed potential differences in PPS among reported injury severity groups (i.e., non-disabling, temporary disabling, permanent disabling, and death) and PPE used at time of incident. Multilevel logistic regression assessed potential associations between PPS and reported injury severity, adjusting for potential confounding factors and correlation of individuals within SDs. [20]

## Results

There are twenty-one county CTE SDs in NJ. The twenty-one counties in NJ had at least one SD submit an injury report between 1998 and 2015. There was a wide range of PPS among CTE SDs, from approximately $6700 to $40,600 during the 1998–2015 school years. When eight CSSD and four CS SDs were included in the analysis, the range of mean PPS increased ($8000 - $66,200). The differences between highest and lowest SD PPS values annually were approximately 60–90%. (Tables 1-3) Average annual PPS trends indicate that inflation-adjusted PPS has generally declined from academic year 1998–1999 to most recent data (Tables 2 and 3). Comparing longitudinal trends between the CTE SDs (Table 2) and CTE together with eight CSSD and four CS SDs (Table 3) suggests that CTE SDs have experienced a greater PPS decline than CSSD and CS SDs. On average, Hudson County CTE SD had the highest PPS (about $24,000) while Hunterdon County CTE SD had the lowest PPS (about $8000). The PPS of Bergen, Camden and Cape May County CTE SDs tended to increase annually, whereas in other CTE SDs, PPS fluctuated over time.

**Table 1:** Average per pupil spending (PPS) of State of New Jersey (NJ), career-technical-vocational education (CTE), county special services district (CSSD), and charter school (CS) school districts (SDs) in Fiscal Years 1998–99 to 2014–15^1^

Type of school districts	Total (n)	Mean ± S.D. mean	Min	Max	Percentile		
					25	50	75
CTE ^a^	21	18,413 ± 5951	6752	40,612	14,766	17,447	22,061
CTE, CSSD and CS^b^	33	23,954 ± 12,917	6752	66,199	14,800	19,014	30,591

^1^Values reported in inflation adjusted, 2010 U.S. dollars

^a^PPS of only 21 CTE school districts from Fiscal Year (FY) 1998–99 to 2014–15 were used for analysis

^b^PPS of 21 VOC and eight CSSD school districts from Fiscal Year 1998–99 to 2014–15, and four CS SDs, were used in analysis

For CS SDs, the available data of each school district were as follows: Academy Charter School, Monmouth County from FY 1998–99 to 2014–15; Charter Technology, Atlantic County from FY 1999–2000 to 2014–15; Camden Academy, Camden County from FY 2001–02 to 2014–15; and, University Academy, Hudson County from FY 2002–03 to 2014–15

**Table 2:** Annual per pupil spending of career-technical-vocational education school districts in the State of New Jersey (*n* = 21 counties, one per county)^a^

School Year	Total (n)	Mean ± S.E. mean	Min	Max	Percentile		
					25	50	75
1998–1999	21	22,084 ± 6627	12,384	40,613	17,714	21,615	26,486
1999–2000	21	22,455 ± 6416	7113	37,883	18,452	22,151	24,910
2000–2001	21	22,986 ± 6050	11,137	37,048	19,258	25,469	26,922
2001–2002	21	20,976 ± 5846	9697	34,560	16,266	21,865	24,311
2002–2003	21	19,837 ± 5475	9923	34,542	16,680	19,992	22,390
2003–2004	21	18,966 ± 5927	8332	33,649	15,959	19,057	21,888
2004–2005	21	18,989 ± 5391	9558	33,270	16,170	18,482	22,628
2005–2006	21	19,026 ± 5780	9160	31,307	15,464	18,150	22,490
2006–2007	21	18,542 ± 5454	8451	33,074	16,294	17,627	18,876
2007–2008	21	17,203 ± 4642	7125	28,996	14,912	16,243	20,416
2008–2009	21	16,983 ± 5210	7466	32,652	13,745	17,233	19,155
2009–2010	21	16,765 ± 6111	6752	30,584	13,014	16,705	19,014
2010–2011	21	15,454 ± 4907	6862	26,285	12,436	15,250	17,444
2011–2012	21	15,472 ± 4734	7469	25,858	12,683	15,341	17,005
2012–2013	21	14,422 ± 3761	7470	22,535	11,978	14,588	16,003
2013–2014	21	14,450 ± 3473	8256	22,306	12,791	13,989	15,859
2014–2015	21	16,939 ± 999	10,292	28,230	13,600	16,266	18,985

^a^Values reported in inflation adjusted, 2010 U.S. dollars

Per pupil spending of each school district was compared in the same state fiscal year (school year)

**Table 3:** Annual (school year) per pupil spending **(**PPS) of career-technical-vocational education (CTE) school districts (SDs), county special services school districts, and charter school (CS) SDs in the State of New Jersey^a^

School Year	Total (n)	Mean ± S.E. mean	Min	Max	Percentile		
					25	50	75
1998–1999	30 (missing = 3)	27,328 ± 11,968	12,384	61,043	18,362	23,593	34,266
1999–2000	31 (missing = 2)	28,559 ± 13,350	7113	65,508	18,452	24,698	39,483
2000–2001	31 (missing = 2)	26,369 ± 9482	11,137	51,272	19,258	25,947	30,592
2001–2002	32 (missing = 1)	25,144 ± 10,690	9697	53,346	16,111	23,319	32,122
2002–2003	33	23,882 ± 11,079	9923	55,051	16,183	20,216	31,756
2003–2004	33	23,455 ± 11,426	8332	53,396	15,959	19,627	32,634
2004–2005	32	25,127 ± 13,379	9558	60,345	16,405	19,791	33,983
2005–2006	33	24,888 ± 13,950	9160	62,884	15,464	19,867	31,307
2006–2007	33	24,565 ± 13,871	8451	61,053	16,218	17,686	33,074
2007–2008	33	23,522 ± 13,923	7125	61,843	14,128	17,146	28,996
2008–2009	33	22,848 ± 12,873	7466	51,641	14,489	17,963	26,922
2009–2010	33	22,974 ± 14,080	6752	62,701	13,532	17,273	30,307
2010–2011	33	22,431 ± 14,958	6862	66,199	12,799	16,367	24,996
2011–2012	33	21,875 ± 13,787	7469	65,254	13,483	16,150	25,858
2012–2013	33	20,991 ± 13,878	7470	65,800	13,184	15,064	22,535
2013–2014	33	20,105 ± 12,501	8256	62,614	12,791	14,842	22,306
2014–2015	33	25,060 ± 2863	10,292	76,653	14,849	17,366	32,252

^a^Values reported in inflation adjusted, 2010 U.S. dollars

PPS among SD compared in the same FY; number of SD varied because missing data from CS SD

The available data of CS SD were: Academy Charter School, Monmouth County from FY 1998–99 to 2014–15; Charter Technology, Atlantic County from FY 1999–2000 to 2014–15; Camden Academy, Camden County from FY 2001–02 to 2014–15; and University Academy, Hudson County from FY 2002–03 to 2014–15

In this analysis, we focused only on PPS of CTE SDs depending on the injury report data. There was higher incidence of injuries among males (72%) than females (28%) (Table 4). The most commonly injured body parts were hands and fingers, and the most commonly reported injury type was cuts/lacerations. [16, 17] Also, 55% of injuries were treated at a hospital/ED compared to 45% being treated at an outpatient clinic. Overall, injury severity was primarily non-disabling (70%), followed by temporarily disabling (30%); only one incident was considered a permanent disability as a finger amputation. The PPE usage was about evenly distributed among use (51%) and no use (49%).

**Table 4:** Summary of injury reports to New Jersey Safe Schools Program state-law based surveillance system, 12/1998–06/2015

Characteristic	Total (n)	Total (%)
Total Injury reports^a^	2066	
Gender		
Male	1475	72.2
Female	569	27.8
Status		
Student	1933	96.3
Staff	75	3.7
Treatment		
Hospital	808	55.0
Doctor	661	45.0
Injured Body Part		
Finger	793	37.6
Hand	218	10.3
Multiple	199	9.4
Eye	158	7.5
Head	87	4.1
Foot	75	3.6
Arm	73	3.5
Face	60	2.8
Back	53	2.5
Ankle	51	2.4
Knee	42	2.0
Other	300	14.2
Injury Type		
Cut/Laceration	815	42.5
Burn	178	9.3
Multiple	165	8.6
Sprain	95	5.0
Bruise/Bump	86	4.5
Fracture	74	3.9
Puncture	72	3.8
Abrasion	50	2.6
Other	381	19.9
Injury Cause		
Struck By	664	34.1
Struck Against	305	15.6
Extreme Temperature	149	7.6
Caught In/Under/Between	134	6.9
Fall (Same Level)	87	4.5
Multiple	63	3.2
Rubbed/Abraded	55	2.8
Other	493	25.3
Severity		
Non-disabling	1327	68.6
Temporarily disabling	606	31.3
Permanent Disability	1	0.1
Personal Protective Equipment (PPE)^b^		
Yes	303	49.3
No	311	50.7

^a^Variables not adding up to total (*N* = 2066) indicated missing data points in injury report;

“Staff” not included in analysis

^b^PPE was not included in the older version of paper report from 1998 to 2003;

yes and no indicate PPE used and no type of PPE used at the time of incidents, respectively

**Fig. 1:**
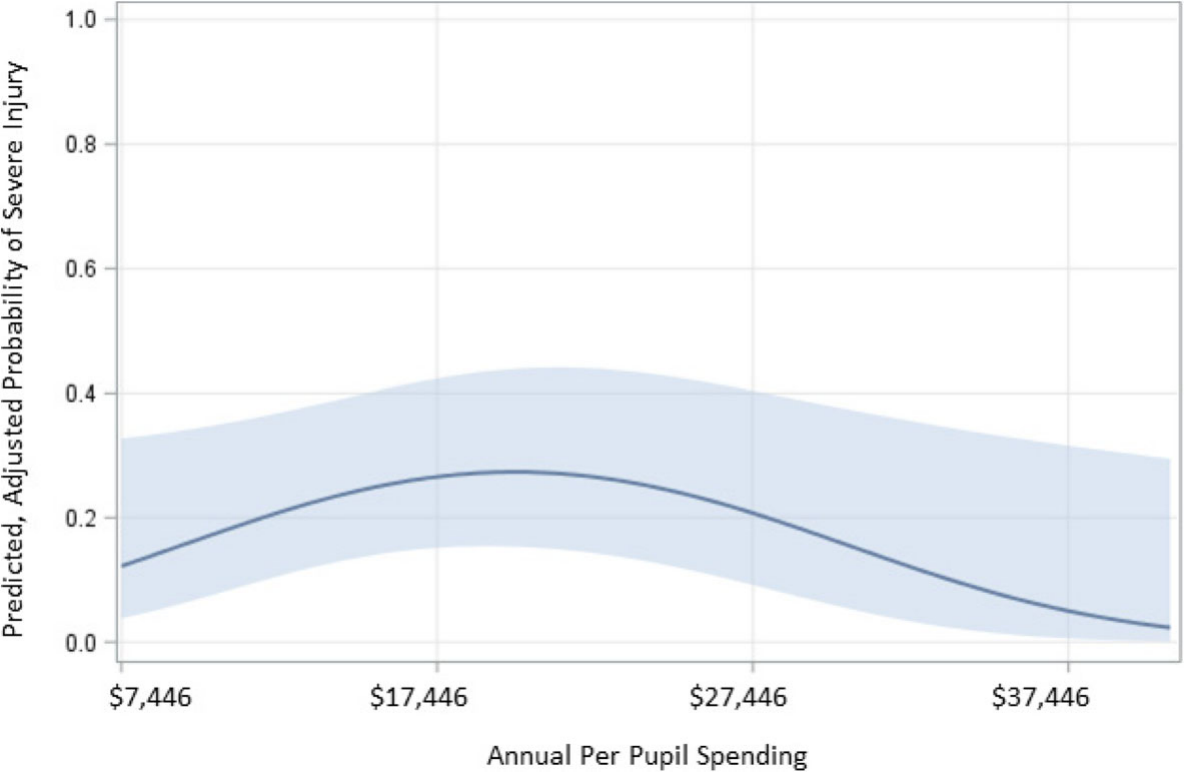
Predicted, adjusted probability of experiencing a temporarily disabling injury by level of school district-level per pupil spending ^a,b^. ^a^ Adjusted for DFG score, location of treatment, and year of injury. ^b^ Annual per pupil spending is inflation adjusted to 2010 U.S. dollars

When stratified by PPS—higher and lower, defined as above and below median at $14,694, respectively—results suggested about two-thirds (68%) of injury incidents were among lower PPS SDs and about one-third (32%) of injury incidents were among higher PPS SDs. Moreover, the percentage injuries considered to be more severe (temporarily disabling) varied by PPS; 26% of all injuries within higher PPS SDs were temporarily disabling compared to 34% of such injuries in lower PPS SDs (Table 5). After adjustment for county DFG score, location of treatment, and year of injury, there was a ‘∩’-shaped relationship between inflation-adjusted PPS and injury severity. The probability of experiencing a more severe injury (Fig. 1) increased with increasing PPS only up to a PPS of $20,000. Among PPS SDs with greater than $20,000 the probability of experiencing a more severe injury decreased with increasing PPS.

**Table 5:** Injury reports to the New Jersey Safe Schools Program state-law based surveillance, 12/1998–06/2015, by per pupil spending (PPS) status

Characteristics	Higher PPS^a^		Lower PPS^b^	
	Number	Percentage	Number	Percentage
Gender				
Male	812	72.1	632	69.3
Female	314	27.9	280	30.7
Treatment (*p* = 0.03)				
Hospital	399	58.0	407	52.3
Doctor	289	42.0	371	47.7
Severity^c^ (*p* = 0.0002)				
Non-disabling	775	72.2	547	64.2
Temporary Disabling	299	27.8	305	35.8
Personal Protective Equipment (PPE) Use^d^ (*p* = 0.86)				
Yes	101	50.0	202	48.9
No	101	50.0	211	51.1

^a^Higher PPS signifies PPS above median, $17,447 (inflation-adjusted 2010 U.S. dollars)

^b^Lower PPS signifies PPS below median, $17,447 (inflation-adjusted 2010 U.S. dollars)

^c^There was one permanent disability reported within the higher PPS group; no deaths reported

^d^Yes and No indicated PPE used and no type of PPE used at the time of incidents, respectively

Statistically significant differences were observed between PPS and nature of injury (results not tabulated). There were significantly higher number of reported injuries, i.e., bruises/bumps (*p* < 0.001), cut/laceration (*p* = 0.03), and other, e.g., pain, seizure, bloody nose, and fainted (*p* = 0.04), among higher PPS than lower PPS SDs. There was significant higher reported incidence of fractures, which are a relatively more severe type of injury, among lower PPS than higher PPS SDs (*p* = 0.001). Also, statistically significant differences were observed between PPS and cause of injury. The injuries among lower PPS SDs were more likely to be caused by fall from elevation (*p* = 0.03); whereas among higher PPS SDs, the injuries were more likely to be caused by caught in/under/between (*p* = 0.01). The injured body part most often reported was ‘other’ (e.g., back, thumb, finger, shoulder and fainting), which was higher among lower PPS than higher PPS SDs (*p* = 0.04). The results also suggested no statistically significant difference between PPE usage and injury among higher and lower PPS SDs.

## Discussion

This study suggested a statistically significant difference between SD-level, per pupil spending and various injury characteristics among NJ youth attending CTE programs. The relatively less severe, non-disabling to temporarily disabling injuries like bruises/bumps were more likely to occur at schools/SDs with higher PPS, conversely more severe injuries like fractures were more likely to occur at schools/SDs with lower PPS.

This study also suggested how for reported injuries there was no statistically significant difference in the location of reported injury treatment, i.e., treated at hospitals/EDs versus treated at outpatient clinics. Components of PPS related to health care facilities and doctor/nurse-student ratio may not be substantially different among schools/SDs with varying total PPS. In addition, in this study, health insurance status may not have been an issue for choosing the location of injury treatment. Reported injuries were usually acquired within school-sponsored CTE programs; thus, costs incurred would potentially be covered by the reporting school/SD and/or the student’s family. Irrespective of who is responsible for paying for treatment, each trip to an ED costs society around $200. [21] Therefore, school-based injury prevention remains warranted.

Furthermore, in a previous report by the U.S. Department of Labor, workplace injuries among the adult population placed burdens on the workers, especially low-wage and immigrant workers, and their families, and have contributed to income inequality, another SES indicator. [22] Moreover, previous studies suggested poor families and minorities with higher health disparities and lower child achievement measures were more likely to attend schools with lower PPS than schools with higher PPS. [23] Further school-based research on SES and race/ethnic disparities in reported CTE injuries and potential impacts on academic performance is needed.

### Limitations

There were several limitations of this study. One limitation was PPS might not be a representative of the real budget of each SD; PPS did not include total revenue, which includes other sources (non-state, private organization, community funding). Other limitations of this study were similar to our prior analyses with DFG scores as an SES indicator. [5] There were no denominator data. This study’s data were reported incidents within CTE programs; there were no data of uninjured students/staff enrolled in CTE program between the school years 1998–2015. Another limitation related to the generalizability of results to the general secondary school/student population. Students enrolled in NJ CTE programs might not represent the overall student population, especially general education students throughout NJ. Also, these students may not generalize to CTE students in other U.S. states, as CTE programs differ among each state. Furthermore, some PPS values were for a specific school within a SD, while some were based on the PPS of the overall SD, which might be only an estimation of the exact amount of PPS among each school reporting injuries. Finally, besides the well-known potential for information bias due to underreporting of data, one other issue related to certain missing or incomplete data fields. However, as of October 2013, reports have been only submitted online to NJSS via Psychdata. Thus, the problem about leaving certain spaces blank as sometimes occurred with the past paper-based injury surveillance system was eliminated. Other analyses compared completeness of reporting factors between the former paper-based and current online reporting system and are reported elsewhere. [24]

This study also had strengths. Data represented injury surveillance over 15 years for the State of NJ. Results inform the literature by specifically examining injuries relating to secondary school students enrolled in CTE programs by PPS as one major SES indicator, and are supported by a previous study [5] but now with 1.5 years more data.

## Conclusions

The NJ Safe Schools Program/NJ Department of Education state law-based online surveillance system for youth/young workers in approved secondary school career-technical-vocational education programs allows examination of potential disparities regarding reportable work-related injuries by per pupil spending or PPS, an indicator of socioeconomic status, as well as by sex or gender, severity, safety training provided and work experience at time of injury. Future research should further explore these associations, and incorporate other attributes of the school environment as well as the presence (or not) of school policies on safety, health, and operations and maintenance of school facilities including classrooms. Moreover, development of enhanced injury prevention trainings and interventions could decrease numbers of reported injuries and thus help both decrease medical expenditures and increase student academic performance.

## Abbreviations

CS: Charter schools
CTE: Career-technical-vocational education
DFG: District factor group score
ED: Emergency department
IRB: Institutional Review Board
NIOSH: National Institute of Occupational Safety and Health of the U.S. Centers for Disease Control and Prevention
NJ SS: New Jersey Safe Schools Program
NJ: State of New Jersey
NJDOE: New Jersey Department of Education
PPE: Personal protective equipment
PPS: Per pupil spending
SD: School district
SES: Socioeconomic status
